# An Interactive Text Messaging Intervention to Improve Adherence to Option B+ Prevention of Mother-to-Child HIV Transmission in Kenya: Cost Analysis

**DOI:** 10.2196/18351

**Published:** 2020-10-02

**Authors:** Yilin Chen, Keshet Ronen, Daniel Matemo, Jennifer A Unger, John Kinuthia, Grace John-Stewart, Carol Levin

**Affiliations:** 1 Department of Global Health, University of Washington Seattle, WA United States; 2 Kenyatta National Hospital Nairobi Kenya

**Keywords:** mHealth, cost analysis, prevention of mother-to-child transmission, antiretroviral therapy adherence, Kenya, mobile phone

## Abstract

**Background:**

Mobile health (mHealth) approaches offer potentially affordable ways to support the care of HIV-infected patients in overstretched health care systems. However, only few studies have analyzed the costs associated with mHealth solutions for HIV care.

**Objective:**

The aim of this study was to estimate the total incremental costs and incremental cost per beneficiary of an interactive SMS text messaging support intervention within a clinical trial.

**Methods:**

The Mobile WAChX trial (NCT02400671) evaluates an interactive semiautomated SMS text messaging intervention to improve adherence to antiretroviral therapy and retention in care among peripartum women infected with HIV in Kenya to reduce the mother-to-child transmission of HIV. Women were randomized to receive one-way versus two-way SMS text messages. Messages were sent weekly, and these messages included motivational and educational content and visit reminders; two-way messaging enabled prompt consultation with the nurse as needed. Microcosting methods were used to collect resource-use data related to implementing the Mobile WAChX SMS text messaging intervention. At 2 sites (Nairobi and Western Kenya), we conducted semistructured interviews with health personnel to identify startup and recurrent activities by obtaining information on the personnel, supplies, and equipment. Data on expenditures and prices from project expense reports, administrative records, and published government salary data were included to estimate the total incremental costs. Using a public provider perspective, we estimated incremental unit costs per beneficiary and per contact during 2017.

**Results:**

The weighted average annual incremental costs for the two-way SMS text messaging group were US $3725 per facility, US $62 per beneficiary, and US $0.85 per contact to reach 115 beneficiaries. For the one-way SMS text messaging group, the weighted average annual incremental costs were US $2542 per facility, US $41 per beneficiary, and US $0.66 per contact to reach 117 beneficiaries. The largest cost shares were for the personnel: 48.2% (US $1794/US $3725) in two-way and 32.4% (US $825/US $2542) in one-way SMS text messaging groups. Costs associated with software development and communication accounted for 29.9% (US $1872/US $6267) of the costs in both intervention arms (US $1042 vs US $830, respectively).

**Conclusions:**

Cost information for budgeting and financial planning is relevant for implementing mHealth interventions in national health plans. Given the proportion of costs related to systems development, it is likely that costs per beneficiary will decline with the scale-up of the interventions.

## Introduction

In 2017, an estimated 180,000 children became infected with HIV [1]. The Joint United Nations Program on HIV and AIDS has set the ambitious target of reducing mother-to-child transmission (MTCT) to 20,000 cases by 2020. A critical factor required to achieve this goal is sustained adherence to antiretroviral therapy (ART) by pregnant and breastfeeding women living with HIV. Suboptimal adherence and virologic suppression in pregnant and postpartum women have been documented in several contexts, and these factors have significantly increased the risk of MTCT and poor maternal outcomes. Thus, development of strategies to support ART adherence and engagement in care has been identified as a priority.

Use of SMS text messaging communication is a promising approach for improving ART adherence in peripartum women. Several studies and meta-analyses have shown that regularly delivered SMS text messages can improve ART adherence and retention in care outside of pregnancy [2-5] and that interactive, two-way SMS text messaging between patients and health care providers is more efficacious than one-way informational messaging [6,7]. Based on these data, SMS text messaging has been identified as a recommended intervention to promote ART adherence by the World Health Organization [8]. In 2016, the Ministry of Health (MOH) of Kenya rolled out a mobile health (mHealth) service “Ushauri” in 105 facilities to manage appointments and deliver standardized messages and reminders to patients with HIV, which was shown to be a promising strategy in improving viral suppression and retention in routine HIV care [9].

While SMS text messaging interventions are supported by policymakers to improve ART adherence, little is known about the value for money of such technologies. In a global survey conducted by the World Health Organization, lack of evidence on economic evaluation was identified as a major barrier to implementation of mHealth solutions in resource-constrained settings [10]. One of the reasons was the limited availability of data on the cost of implementing SMS text messaging interventions [11,12]. Only 1 study has performed a cost analysis of one-way SMS text messaging promoting the availability of HIV self-testing kits in Kenyan clinics [13]. We found no cost analysis data on implementing two-way SMS text messaging interventions during our literature review. While the Kenyan MOH is currently planning nationwide scale-up of mHealth interventions to meet universal health coverage (UHC) goals [9], it is essential to understand the costs for budgeting purposes and for designing efficient and affordable programs that can be scaled nationally [14]. We therefore conducted a cost analysis of one-way and two-way SMS text messaging interventions (vs control) in the Mobile WAChX study, which is a randomized controlled trial of semiautomated SMS text messaging interventions to improve ART adherence and retention in care in peripartum women at 6 facilities in Nairobi and Western Kenya [15]. The primary objective of this study was to estimate the total incremental costs, incremental cost per beneficiary, and cost per contact associated with the SMS text messaging interventions. The secondary objective was to estimate the costs of alternative implementation and usage scenarios for future scale-up of the interventions.

## Methods

### Study Design

The Mobile WAChX trial (NCT02400671) evaluates one-way versus two-way communication versions of a semiautomated SMS text messaging intervention to improve ART adherence and retention in care in peripartum women at 6 facilities in Nairobi and Western Kenya. One-way SMS text messaging consisted of weekly automated motivational and educational SMS text messages and clinic visit reminders. Participants randomized to the two-way SMS text messaging arm additionally had the capability of communicating with a nurse through the SMS text messaging system. Participants randomized to the control arm received no SMS text messages (standard of care).

The study procedures for the randomized controlled trial are described in detail in a previous paper [15]. For costing analysis, convenience sampling was used to collect the cost data from 2 of the 6 sites: an urban health center in Nairobi (facility A) and a rural subcounty hospital in Western Kenya (facility B). We used a provider perspective to analyze the incremental financial and economic costs of implementing Mobile WAChX alongside existing maternal and child health (MCH) services from January 2017 to December 2017, as well as the average incremental cost per beneficiary and average incremental cost per contact. Beneficiaries are service users who accessed Mobile WAChX SMS text messaging communication, including automated messages and personalized messaging with nurses, through the Mobile WAChX system. The number of contacts is equal to the number of total messages sent successfully to the beneficiaries. Data on the number of beneficiaries and contacts at each facility were extracted from ongoing project monitoring and evaluation reports and electronic databases of SMS text messages.

### Data Collection Method

We used an activity-based ingredients approach to identify all activities undertaken to deliver the Mobile WAChX project. Activities included intervention planning, project partner sensitization, staff training, system development, and delivery of SMS text messages. After identifying all intervention-related activities, we quantified the resources used and valued these by using the best available data on salaries and commodity prices. We used a combination of data collection methods to collect primary resource-use and cost data, including obtaining prices from project expense reports, administrative records, and published government salaries, and conducting semistructured interviews with facility-based health workers and project administrators. Time-motion studies were conducted in both facilities to record staff time spent on intervention activities (eg, recruiting, screening, and registering participants, sending SMS text messages to users). Data collection was conducted between October 2017 and January 2018.

### Cost Categories

We organized cost data into one-time fixed costs and variable costs (Table 1). Fixed costs were categorized according to the following activities: intervention planning, preparation of intervention sites, development of the Mobile WAChX SMS text messaging management system, initial training, and sensitization meetings with facility staff and Kenyan MOH officials. All fixed costs were used for one-time start-up activities, where we assumed a 5-year useful life. Variable costs included recurrent costs, which were required to sustain the intervention. They were divided into mutually exclusive input categories for personnel, communication, equipment, and overhead. Personnel costs included salaries, benefits, and allowances of facility-based health workers as well as of staff in charge of personnel supervision and coordination. A study nurse and a retention officer delivered Mobile WAChX-related activities. Shared facility costs included a study coordinator and a data manager who were responsible for supervising all the 6 sites and some shared communications costs such as the costs of developing a Mobile WAChX system and cost of the system-hosting platform. We allocated these shared facility costs based on the annual share of clients served at the facility, as a percentage of the total annual number of clients served for all 6 facilities. In addition to the cost of a system-hosting platform, other communication costs included cost of internet data bundles for using the internet-based Mobile WAChX system, airtime cost for making patient follow-up phone calls, costs of sending SMS text messages to users, and short code toll-free numbers used by participants to deliver messages to the nurse. Equipment costs (mobile phones, laptops, and furniture) were annuitized over the useful life of 10 years by using a discount rate of 3%. All costs in this evaluation were expressed in USD, using the official exchange rate of 1 USD to 103.25 Kenyan Shillings (2017 exchange rate). In addition, research time and other research costs were removed from the costing analysis. International staff time was also excluded to better reflect the costs of the program when implemented and scaled locally.

**Table 1:** Activity and input cost categories and description.

Cost categories, subcategories			Description	
**Fixed costs**				
	Planning/microplanning			Planning activities for project implementation during the start-up period.
	System development			Resources and inputs to design the Mobile WAChX system and activities to collaborate with a local mobile technology company to obtain SMS text messaging packages for participants.
	Initial training			Expenses for conducting 2 training workshops during the start-up period for all project staff, including development of relevant training materials.
	Sensitization			Stakeholder workshops and activities at facility level.
**Variable costs**				
	**Personnel**			Value of personnel time
		Service delivery		Activities for delivering the Mobile WAChX intervention, such as recruiting participants, screening and registering participants, and sending SMS text messages to users.
		Personnel supervision and coordination		Meetings to supervise staff and coordinate and monitor implementation of activities at all sites.
	Communication			Resources and inputs to deliver SMS text messages to participants, including data bundles for internet, an online platform for hosting the Mobile WAChX system, airtime cost for phone calls, and SMS text messaging cost.
	Equipment			Investments that last longer than 1 year, including mobile phones, laptops, and furniture.
	Overhead			Clinic collaboration fee and indirect costs.

### Data Analysis

We developed an Excel-based model (Microsoft Excel version 15.28, Redmond) to estimate total incremental costs and incremental unit costs. The sum of all the activity cost categories reflects all the resources required to deliver the Mobile WAChX intervention. All activities were mutually exclusive, thereby avoiding double counting. We used project output data on the number of beneficiaries per month per facility and number of messages sent by the system and nurses per month. These data were collected as part of the Mobile WAChX monitoring and evaluation strategy. We first estimated the total incremental cost for each facility to deliver the Mobile WAChX intervention and divided this by the number of women receiving the intervention to determine the cost per beneficiary. We also estimated the cost per contact, defined as the total cost divided by the total number of messages sent to participants during 2017, for each facility. We analyzed the costs of one-way and two-way SMS text messaging interventions separately. We also estimated the average weighted costs for both facilities by using project output data on the number of beneficiaries per facility.

### Scenario Analysis

In addition to estimating intervention costs, we estimated 3 scenarios. The first scenario was to estimate the cost when the two-way Mobile WAChX intervention was implemented in all 6 facilities in this project. In this scenario, the same intervention and personnel were applied to every facility where they shared most start-up costs from system development, cost of system-hosting platform, as well as personnel supervision and coordination costs. The number of participants was the total number of participants receiving intervention in all 6 sites. The second scenario was to estimate a more typical scenario where the MOH of Kenya supports these activities after the pilot phase. We applied the MOH salary scale to service delivery health workers in the current project. Finally, we calculated the incremental cost effectiveness ratios (ICERs) of comparing two-way SMS text messages to no intervention in the 2 facilities. Due to unavailable efficacy data in the Mobile WAChX trial, we used clinical outcome results from a similar randomized controlled trial, which assessed whether two-way SMS text messaging interventions improved plasma HIV-1 viral RNA load suppression at 12 months in 3 clinics in Kenya [7]. The efficacy of mobile SMS text messaging intervention for medication adherence was obtained from a meta-analysis study [16]. Other inputs were using project output data and our analyses.

## Results

### Project Output

Among the 152 HIV-infected women in facility A who received the Mobile WAChX intervention, 76 (50.0%) were randomized to the two-way SMS text messaging group and the other 76 (50.0%) were randomized to the one-way SMS text messaging group. A total of 80 women participated in facility B, of whom 39 were randomized to the two-way SMS text messaging intervention group and 41 were randomized to the one-way SMS text messaging intervention group (Table 2). The beneficiaries in these 2 facilities accounted for 27.7% (152/548) and 14.6% (80/548) of the total number of project beneficiaries at the time of data collection in facilities A and B, respectively. The total number of automated SMS text messages from the system sent to beneficiaries in the 2 facilities in 2017 was 6924 in the two-way SMS text messaging intervention group and 7318 in the one-way SMS text messaging intervention group. The nurses in the 2 facilities sent 1386 personalized messages in response to two-way participant messages during 2017.

**Table 2:** Summary of the beneficiaries and the total points of contact by health facility in 2017.

Health facility	Two-way SMS text messaging intervention			One-way SMS text messaging intervention		
	Beneficiaries, n=115, n (%)	Total automated SMS text messages, n=6924, n (%)	Total nurse SMS text messages, n=1386, n (%)	Beneficiaries, n=117, n (%)	Total automated SMS text messages, n=7318, n (%)	Total nurse SMS text messages, n=0, n (%)
Facility A (Urban health center)	76 (66.1)	4425 (63.9)	993 (71.6)	76 (65.0)	4604 (62.9)	0 (0)
Facility B (Rural subcounty hospital)	39 (33.9)	2499 (36.1)	393 (28.4)	41 (35.0)	2714 (37.1)	0 (0)

### Total Costs and Unit Costs

**Figure 1:**
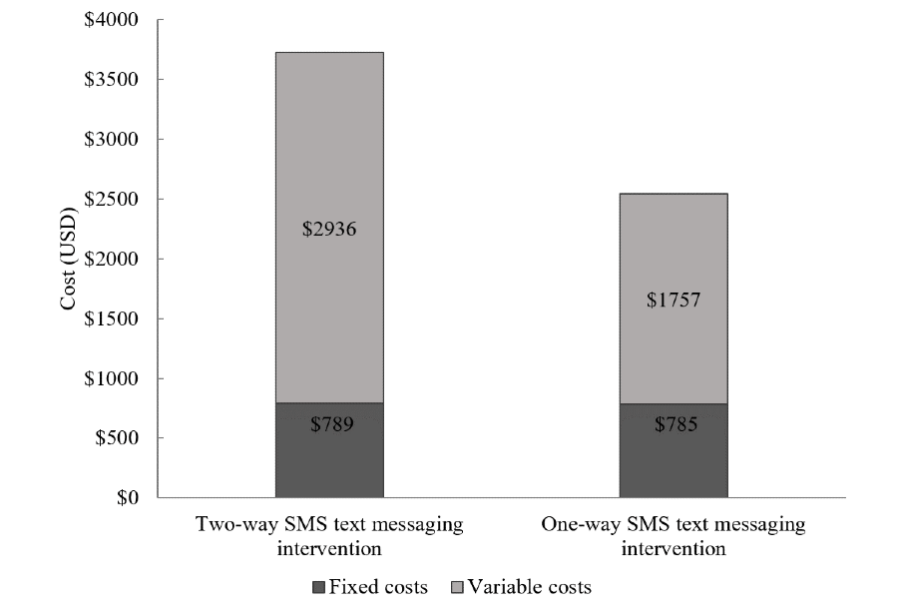
Total annual incremental costs by fixed and variable costs.

**Figure 2:**
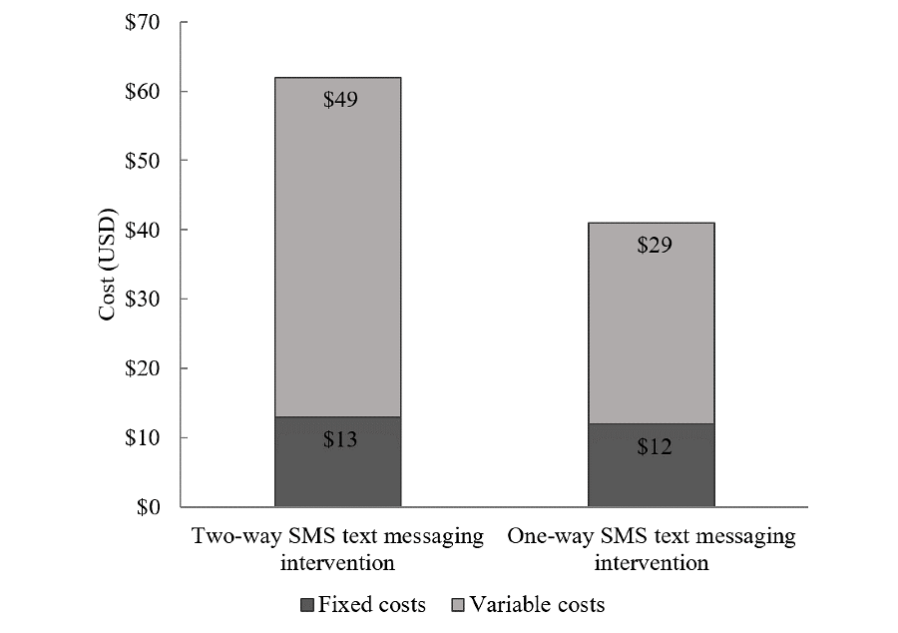
Cost per beneficiary by fixed and variable costs.

**Figure 3:**
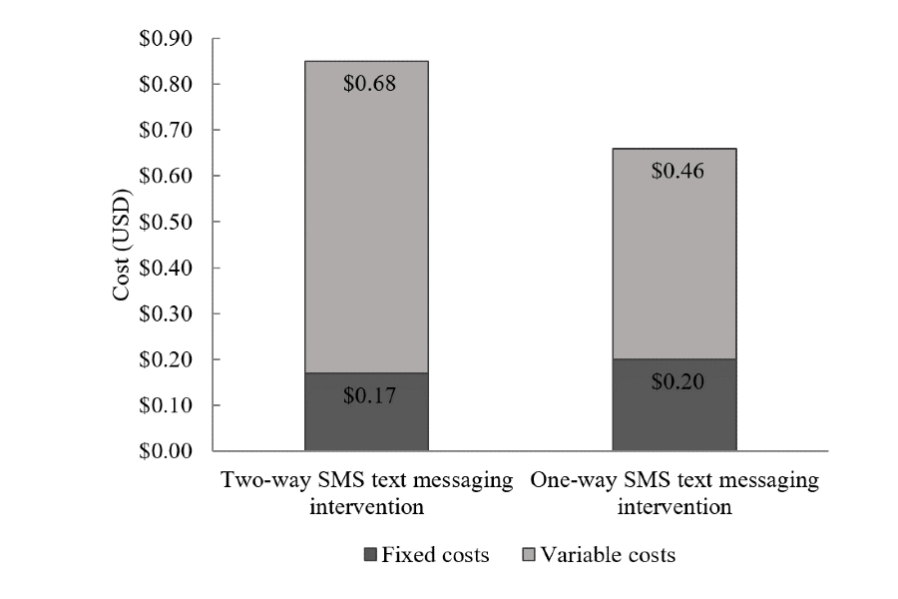
Cost per contact by fixed and variable costs.

Table 3 summarizes the weighted average total annual incremental costs and incremental unit costs for beneficiaries in 2017. The weighted average total cost of the Mobile WAChX intervention was estimated at US $3725 for the two-way SMS text messaging group and US $2542 for the one-way SMS text messaging group. Fixed costs were US $2936 for two-way SMS text messaging and US $1757 for one-way SMS text messaging intervention groups (Figure 1), while variable costs were similar across the 2 groups (US $789 and US $785, respectively). The weighted average cost per beneficiary for the two-way group was higher than that for the one-way SMS text messaging intervention group (US $62 and US $41, respectively) (Figure 2). In addition, the weighted average cost per contact was estimated at US $0.85 for the two-way and US $0.66 for the one-way SMS text messaging intervention group (Figure 3). The detailed annual incremental costs and cost per beneficiary by input categories for each facility and the weighted average estimates are presented in Multimedia Appendix 1, Multimedia Appendix 2, and Multimedia Appendix 3.

**Table 3:** Weighted average total annual incremental costs and unit costs for beneficiaries.

Intervention group	Total costs and unit costs (USD)		
	Total annual cost	Cost per beneficiary	Cost per contact
Two-way SMS text messaging	$3725	$62	$0.85
One-way SMS text messaging	$2542	$41	$0.66

### Cost Profiles

**Figure 4:**
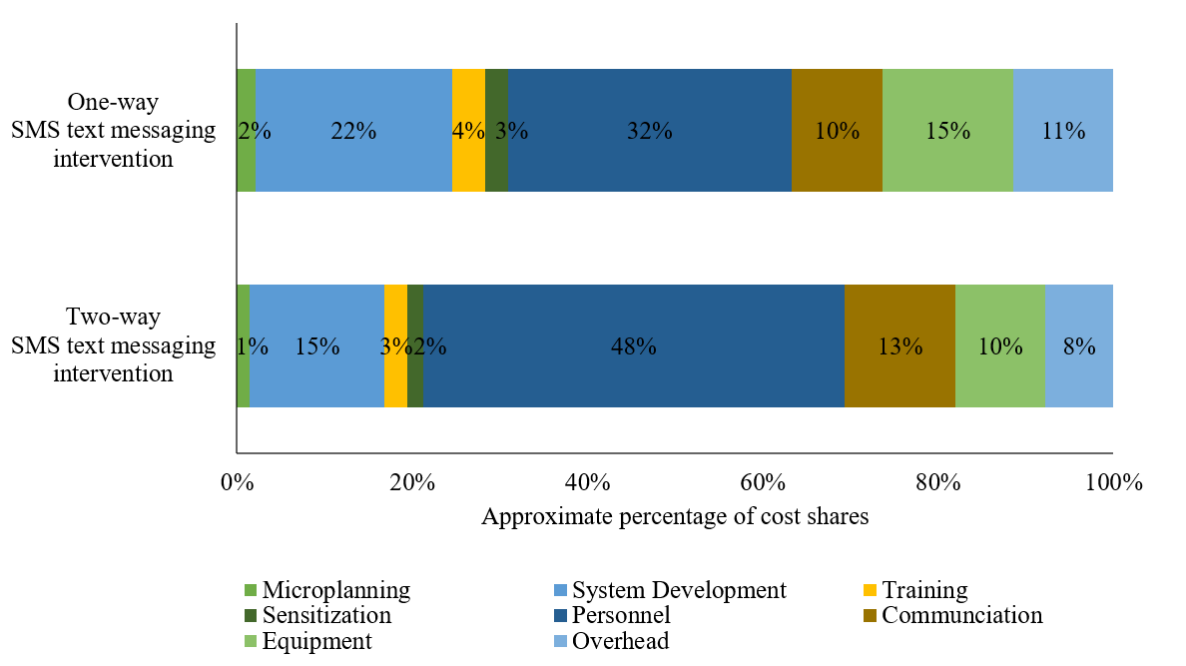
Cost shares by input categories for all costs.

**Figure 5:**
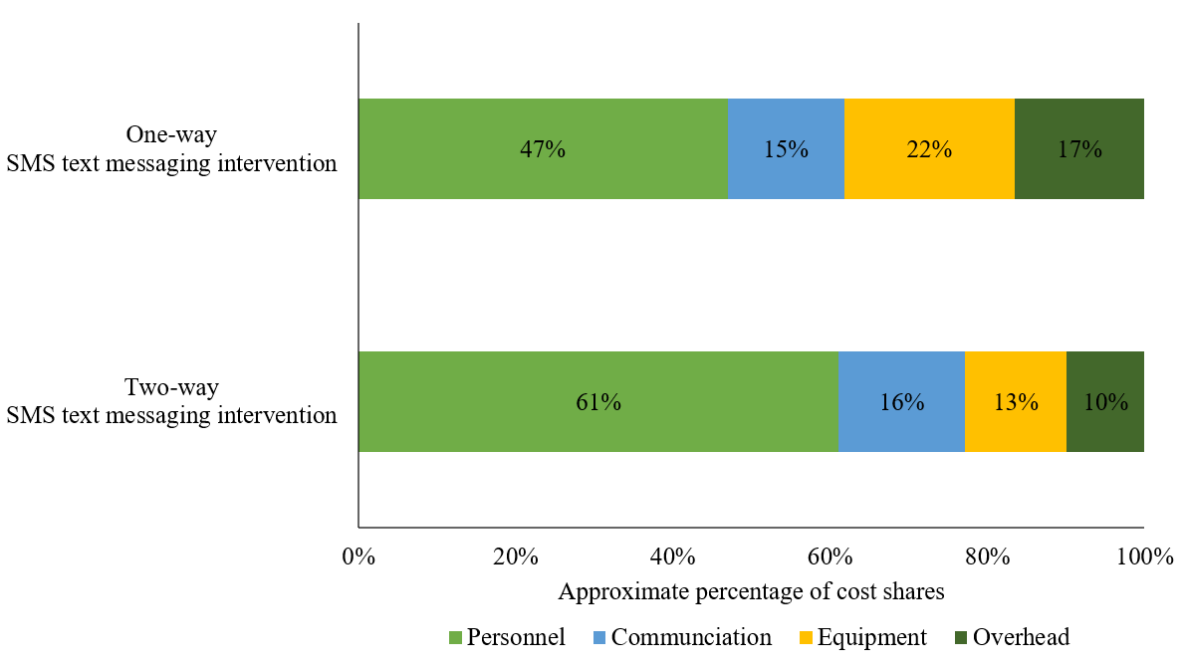
Cost shares for variable input categories only.

Figure 4 presents the cost shares by activity and input cost categories. Personnel cost accounted for the largest share of the total costs. The personnel cost in the one-way SMS text messaging group (US $825/US $2542, 32.4%) was lower than that of the two-way SMS text messaging group (US $1794/US $3725, 48.2%), as one-way messaging required no personnel time responding to messages. The second largest share of the total costs was related to software development of the SMS text messaging management system (two-way: US $573/US $3725, 15.4% vs one-way: US $569/US $2542, 22.4%) and communication (two-way: US $470/US $3725, 12.6% vs one-way: US $261/US $2542, 10.3%) such as flat rate fees for internet usage and mobile phone minutes. In the two-way SMS text messaging group, an estimated 10.2% (US $381/US $3725) of the total costs was used for purchasing equipment and mobile devices to set up the intervention site in the facility and to implement the Mobile WAChX intervention. Overhead costs accounted for 7.8% (US $291/US $3725) of the total costs in the two-way SMS text messaging group, in the form of a clinic collaboration fee to use a clinic room for study activities (US $48 per month). Two trainings were conducted at the beginning of the project to teach nurses and retention officers how to use the Mobile WAChX system and orient them to implement the intervention, which costs US $98 per facility (3% of the total costs). The remaining budget was allocated to sensitization (US $64) and microplanning (US $54), which accounted for 3.2% (US $118/US $3725) of the total costs. The main cost drivers were similar in both two-way and one-way SMS text messaging intervention groups. The variable cost shares by input categories are shown in Figure 5. The majority of the total costs were related to personnel in both two-way and one-way SMS text messaging intervention groups (US $1794/US $2936, 61.1% vs US $825/US $1757, 46.9%, respectively), followed by communication (US $470/US $2936, 16.0% vs US $261/US $1757, 14.9%, respectively), equipment (US $381/US $2936, 13.0% vs US $381/US $1757, 21.7%, respectively), and overhead (US $291/US $2936, 9.9% vs US $291/US $1757, 16.5%, respectively).

### Sensitivity and Scenario Analysis

Cost estimates in the 2 scenarios compared to the baseline scenario estimates reflected in Table 3 are presented in Multimedia Appendix 4. Expanding the two-way SMS text messaging intervention to all 6 facilities would have the effect of decreasing the estimated cost per beneficiary by 31% (from US $62 to US $43; US $19/US $62) and cost per contact by 26% (from US $0.85 to US $0.63; US $0.22/US $0.85). When building on scenario 1 and replacing research project health worker salaries with the more typical salaries for MOH staff, the cost per beneficiary decreased by 50% (from US $62 to US $31, US $31/US $62) and the cost per contact decreased by 46% (from US $0.85 to US $0.46, US $0.39/US $0.85).

The ICERs of comparing two-way SMS text messaging versus no intervention in the 2 facilities and the input parameters are summarized in Multimedia Appendix 5 and Multimedia Appendix 6, respectively. According to published RCT and meta-analysis estimates, implementing two-way SMS text messaging intervention was estimated to have suppressed viral load in 11 patients and achieved medication adherence among 15 patients, with total annual incremental costs of US $7084 [7,16]. Thus, the ICERs were US $644 per viral load suppression and US $472 per medication adherence.

## Discussion

This is the first study to estimate the costs of an mHealth intervention to promote prevention of MTCT (PMTCT)-ART adherence among peripartum women. Previous studies have shown that SMS text messaging interventions have a positive impact on ART adherence and maternal and neonatal health outcomes in low-and-middle income settings [4,17-20]. However, data regarding the programmatic costs of implementing these mHealth interventions are scant [4,18,21]. We estimate the average total incremental costs for 1 year of project implementation to be US $3725 per facility, US $62 per beneficiary, and US $0.85 per contact for the two-way SMS text messaging intervention. For the one-way SMS text messaging intervention, the average total incremental costs are US $2542 per facility, US $41 per beneficiary, and US $0.66 per contact. The higher costs for the two-way SMS text messaging group is due to the personnel time spent responding to SMS text messages, which is not provided in the one-way SMS text messaging group.

Only a few studies have provided the cost estimates of mHealth interventions, including SMS text messaging interventions, in low-income countries. The MAMA program was initiated in 2012 in South Africa to enhance the utilization of MCH services among pregnant and postpartum women by sending registered users SMS text messages twice per week. The estimated program costs over 5 years was US $1.2 million, 17% of which was incurred by costs on program development and 31% on SMS text message delivery costs [22]. The Chipatala cha pa foni (CCPF) project consisted of a toll-free hotline and a mobile phone–based tips and reminders service seeking to improve maternal and neonatal health in Balaka District, Malawi [11]. The tips and reminders service was a one-way messaging system, wherein community health workers sent weekly text or voice messages to participants. Service users could call the hotline as well. The costs during a 2-year period (2011-2012) were estimated to be US $29.33 per user and US $4.33 per successful contact. The ReMiND (Reducing Maternal and Newborn Deaths) Project was designed to improve the quality of counseling of community health workers in India [12]. The mHealth app was implemented through 259 accredited social health activist workers. The total program costs over 3 years (2012-2015) were estimated at US $191,894, with US $20.50 per registered woman. Labor costs accounted for 57% of the total costs, followed by mobile phone purchases and data/internet charges (6%). The annual number of beneficiaries were 9798 and 9390 in CCPF and ReMiND projects, respectively. Both CCPF and ReMiND studies had lower cost per user compared to our study because of the higher number of beneficiaries. Moreover, the ReMiND project did not involve any SMS text message exchange between patients and health workers, which decreases data usage and airtime-related costs. In addition, we found that the higher costs for the two-way SMS text messaging intervention largely resulted from higher personnel costs. As previous studies have demonstrated that two-way SMS text messaging interventions are likely to be more efficacious than one-way SMS text messaging interventions, the higher cost may contribute to health service utilization and better health outcomes. Compared with the cost estimates in the CCPF project, our estimated costs per contact were much lower, both in the two-way and one-way SMS text messaging interventions (US $0.85 and US $0.66, respectively), thereby suggesting overall higher utilization of messaging interventions in our study.

Personnel costs accounted for the largest share of the total costs in our project (US $1794/US $3725, 48.2% in two-way and US $825/US $2542, 32.4% in one-way SMS text messaging intervention groups), followed by software development of the SMS text messaging management system and communication costs. Our results are consistent with findings from previous studies that labor costs for delivering other SMS text messaging interventions were the main drivers of the total program costs, followed by SMS text messaging program development [11,12,22]. How could we potentially reduce system development and communication-related costs as well as personnel costs? Our scenario analysis results suggest that the costs would be reduced significantly by expanding the two-way intervention to more beneficiaries (>500), with cost per beneficiary decreasing from US $62 to US $43 and cost per contact from US $0.85 to US $0.63. Although recurrent communication costs such as airtime and cost of SMS text messages would increase due to increased number of beneficiaries and messages exchanged, the total unit cost would eventually go down because the fixed start-up costs associated with system development and the system-hosting platform would be shared across a larger number of beneficiaries. In addition, the shared program costs for personnel supervision and coordination costs (>60% of total costs) would be allocated across a higher number of beneficiaries. In the second scenario analysis, we explored what a more typical government-sponsored program might cost. Keeping the activities the same, if we replaced health worker salaries in our project with the more typical salaries for MOH staff, the unit costs would go down further to US $31 per beneficiary and US $0.46 per contact. This means moving from a research-focused project to part of a routine government-supported program will result in much lower cost per beneficiary through greater economies of scale and through a more typical mode of service delivery and supervision.

Our findings provide important costing information for budgeting and financial planning for implementing mHealth interventions to achieve UHC in Kenya. mHealth interventions, as a part of the broader eHealth interventions, may become transformational strategies in addressing public health challenges and striving toward UHC in Kenya. This need has also been reaffirmed by the Kenya’s Health Policy (2014-2030), National eHealth Policy (2016-2030), Information Technology Master Plan, and the Health Bill [7-10]. While the Kenyan MOH is currently planning for nationwide scale-up of mHealth systems, affordability cannot be ignored. Assuming 80% mobile phone penetration and that 59,000 women were offered PMTCT services in Kenya [23,24], we could estimate that scaling two-way SMS text messaging intervention to increase adherence would result in total spending of US $1.46 million with the average cost per beneficiary of US $31. This is likely to be affordable as it only comprises 3% of the 2015-2016 PMTCT budget of US $46 million. In addition, our estimated ICERs per patient with viral load suppressed (US $644) and per patient achieving medication adherence (US $472) were both much lower than the 2017 gross domestic product per capita in Kenya (US $1568). Our study showed that, with expanded coverage, the total unit cost will decrease significantly due to shared fixed start-up costs associated with system development and the system-hosting platform. In the future, market forces and private sector could also be harnessed to achieve affordability and sustainability [11]. With the increasingly active participation of the private sector in public health in Kenya, public-private partnership could be explored to leverage the infrastructure and resources of private sectors to help Kenya achieve UHC. For example, there are innovative ways to lower the costs of communication such as by obtaining discounted SMS text messaging packages from local or foreign telecommunication companies.

To our knowledge, this is the first study to estimate the costs of an mHealth intervention targeting PMTCT and MCH in Kenya. Our study has limitations. First, we did not include the labor costs of international collaborators who contributed to the program design and installation and other start-up activities such as work planning meetings. Second, we had to make assumptions about the allocation of communication costs to research and implementation activities, which may influence the total implementation costs. Third, we did not allocate equipment costs to research activities, which may lead to overestimation of the total implementation costs. Equipment such as laptops and phones were mainly used for service delivery, and we do not have detailed information on how much equipment was used to support the research-related activities. Therefore, the evidence should be used with the consideration that after taking into account the equipment costs allocated to research activities, the total incremental cost of implementing SMS text messaging interventions would be lower than the estimates in this study.

In conclusion, this study fills the knowledge gap on the costs of mHealth approaches for improving PMTCT-ART adherence among pregnant women in Kenya. When operating at scale, there may be opportunities to reduce the costs per beneficiary. As the Mobile WAChX intervention is scaled up, further research is needed to understand the economic impact from different perspectives, including cost-utility analyses to assess the value for money compared with alternative approaches to improve women’s clinical outcomes and adherence to HIV treatment as part of PMTCT services.

## Appendix

Multimedia Appendix 1 Annual incremental costs and cost per beneficiary by activity at facility A.

Multimedia Appendix 2 Annual incremental costs and cost per beneficiary by activity at facility B.

Multimedia Appendix 3 Weighted average annual incremental costs and cost per beneficiary by activity at facility A and facility B.

Multimedia Appendix 4 Sensitivity and scenario analysis: total annual costs and unit costs for beneficiaries.

Multimedia Appendix 5 Incremental cost-effectiveness ratios of two-way SMS versus no intervention in viral load suppression and adherence.

Multimedia Appendix 6 Input parameters for incremental cost-effectiveness ratios of two-way SMS versus no intervention in viral load suppression and adherence.

## Abbreviations

ART: antiretroviral therapy
CCPF: Chipatala cha pa foni
ICER: incremental cost effectiveness ratio
MCH: maternal and child health
mHealth: mobile health
MOH: Ministry of Health
MTCT: mother-to-child transmission
PMTCT: prevention of mother-to-child transmission
ReMiND: Reducing Maternal and Newborn Deaths
UHC: universal health coverage
