# Supplementary material for: An Interactive Text Messaging Intervention to Improve Adherence to Option B+ Prevention of Mother-to-Child HIV Transmission in Kenya: Cost Analysis
Source: JMIR Mhealth Uhealth. 2020 Oct 2;8(10):e18351. doi: 10.2196/18351 (PMC7568211; doi:10.2196/18351)
Supplement: Multimedia Appendix 1 [file mhealth_v8i10e18351_app1.docx]

**Multimedia Appendix 1.** Annual incremental costs and cost per beneficiary by activity at facility A.

| **Total Costs and Unit Costs** | | | | | | |
| --- | --- | --- | --- | --- | --- | --- |
|  | **Total annual cost** | | **Cost per beneficiary** | | **Cost per contact** | |
|  |  |  |  |  |  |  |
|  | **One-way (N=76)** | **Two-way (N=76)** | **One-way (N=76)** | **Two-way (N=76)** | **One-way (N=4604)** | **Two-way (N=5418)** |
| ***Fixed costs*** | | | | | | |
| Microplanning | $61.29 | $61.29 | $0.81 | $0.81 | $0.01 | $0.01 |
| System Development | $682.11 | $682.11 | $8.98 | $8.98 | $0.15 | $0.13 |
| Initial Training | $116.87 | $116.87 | $1.54 | $1.54 | $0.03 | $0.02 |
| Sensitization | $64.38 | $64.38 | $0.85 | $0.85 | $0.01 | $0.01 |
| ***Sub-total*** | ***$924.65*** | **$924.65** | ***$12.17*** | ***$12.17*** | ***$0.20*** | ***$0.17*** |
| ***Variable costs*** | | | | | | |
| *Personnel* | | | | | | |
| Service delivery cost | $76.45 | $1131.44 | $1.01 | $14.89 | $0.02 | $0.21 |
| Personnel supervision and coordination | $838.76 | $838.76 | $11.04 | $11.04 | $0.18 | $0.15 |
| *Communication (internet costs, mobile phone minutes, etc)* | | | | | | |
| Data bundles & shared platform | $179.35 | $421.13 | $2.36 | $5.54 | $0.04 | $0.08 |
| Airtime & SMS | $97.14 | $103.52 | $1.28 | $1.36 | $0.02 | $0.02 |
|  | | | | | | |
| Equipment | $401.15 | $401.15 | $5.28 | $5.28 | $0.09 | $0.07 |
| Overhead/Clinic collaboration fee | $290.56 | $290.56 | $3.82 | $3.82 | $0.06 | $0.05 |
| ***Sub-total*** | ***$1883.40*** | ***$3186.56*** | ***$24.78*** | ***$41.93*** | ***$0.41*** | ***$0.59*** |
| **Summary** | ***$2808.05*** | ***$4111.21*** | ***$36.95*** | ***$54.09*** | ***$0.61*** | ***$0.76*** |
