# Supplementary material for: An Interactive Text Messaging Intervention to Improve Adherence to Option B+ Prevention of Mother-to-Child HIV Transmission in Kenya: Cost Analysis
Source: JMIR Mhealth Uhealth. 2020 Oct 2;8(10):e18351. doi: 10.2196/18351 (PMC7568211; doi:10.2196/18351)
Supplement: Multimedia Appendix 2 [file mhealth_v8i10e18351_app2.docx]

**Multimedia Appendix 2.** Annual incremental costs and cost per beneficiary by activity at facility B.

| **Total Costs and Unit Costs** | | | | | | |
| --- | --- | --- | --- | --- | --- | --- |
|  | **Total annual cost** | | **Cost per beneficiary** | | **Cost per contact** | |
|  |  |  |  |  |  |  |
|  | **One-way  (N=41)** | **Two-way (N=39)** | **One-way (N=41)** | **Two-way (N=39)** | **One-way (N=2714)** | **Two-way (N=2892)** |
| ***Fixed costs*** | | | | | | |
| Microplanning | $40.93 | $40.93 | $1.00 | $1.05 | $0.02 | $0.01 |
| System Development | $359.01 | $359.01 | $8.76 | $9.21 | $0.13 | $0.12 |
| Initial Training | $61.75 | $61.75 | $1.51 | $1.58 | $0.02 | $0.02 |
| Sensitization | $64.38 | $64.38 | $1.57 | $1.65 | $0.02 | $0.02 |
| ***Sub-total*** | ***$526.07*** | **$526.07** | ***$12.83*** | ***$13.49*** | ***$0.19*** | ***$0.18*** |
| ***Variable costs*** | | | | | | |
| *Personnel* | | | | | | |
| Service delivery cost | $215.84 | $1009.90 | $5.26 | $25.89 | $0.08 | $0.35 |
| Personnel supervision and coordination | $441.45 | $441.45 | $10.77 | $11.32 | $0.16 | $0.15 |
| *Communication (internet costs, mobile phone minutes, etc)* | | | | | | |
| Data bundles & shared platform | $171.47 | $298.72 | $4.18 | $7.66 | $0.06 | $0.10 |
| Airtime & SMS | $61.97 | $63.37 | $1.51 | $1.62 | $0.02 | $0.02 |
|  | | | | | | |
| Equipment | $342.33 | $342.33 | $8.35 | $8.78 | $0.13 | $0.12 |
| Overhead/Clinic collaboration fee | $290.56 | $290.56 | $7.09 | $7.45 | $0.11 | $0.10 |
| ***Sub-total*** | ***$1523.62*** | ***$2446.33*** | ***$37.16*** | ***$62.73*** | ***$0.56*** | ***$0.85*** |
| **Summary** | ***$2049.69*** | ***$2972.40*** | ***$49.99*** | ***$76.22*** | ***$0.76*** | ***$1.03*** |
