# Supplementary material for: An Interactive Text Messaging Intervention to Improve Adherence to Option B+ Prevention of Mother-to-Child HIV Transmission in Kenya: Cost Analysis
Source: JMIR Mhealth Uhealth. 2020 Oct 2;8(10):e18351. doi: 10.2196/18351 (PMC7568211; doi:10.2196/18351)
Supplement: Multimedia Appendix 3 [file mhealth_v8i10e18351_app3.docx]

**Multimedia Appendix 3.** Weighted average annual incremental costs and cost per beneficiary by activity at facility A and facility B.

| **Total Costs and Unit Costs** | | | | | | |
| --- | --- | --- | --- | --- | --- | --- |
|  | **Total annual cost** | | **Cost per beneficiary** | | **Cost per contact** | |
|  |  |  |  |  |  |  |
|  | **One-way (N=117)** | **Two-way (N=115)** | **One-way (N=117)** | **Two-way (N=115)** | **One-way (N=117)** | **Two-way (N=115)** |
| ***Fixed costs*** | | | | | | |
| Microplanning | $54.15 | $54.38 | $0.87 | $0.89 | $0.01 | $0.01 |
| System Development | $568.89 | $572.54 | $8.90 | $9.05 | $0.14 | $0.13 |
| Initial Training | $97.56 | $98.18 | $1.53 | $1.55 | $0.02 | $0.02 |
| Sensitization | $64.38 | $64.38 | $1.10 | $1.12 | $0.02 | $0.02 |
| ***Sub-total*** | ***$784.98*** | ***$789.48*** | ***$12.40*** | ***$12.61*** | ***$0.20*** | ***$0.17*** |
| ***Variable costs*** | | | | | | |
| *Personnel* | | | | | | |
| Service delivery cost | $125.30 | $1090.22 | $2.50 | $18.62 | $0.04 | $0.26 |
| Personnel supervision and coordination | $699.53 | $704.02 | $10.94 | $11.13 | $0.18 | $0.15 |
| *Communication (internet costs, mobile phone minutes, etc)* | | | | | | |
| Data bundles & shared platform | $176.59 | $379.62 | $3.00 | $6.26 | $0.05 | $0.09 |
| Airtime & SMS | $84.81 | $89.90 | $1.36 | $1.45 | $0.02 | $0.02 |
|  | | | | | | |
| Equipment | $380.54 | $381.20 | $6.35 | $6.47 | $0.10 | $0.09 |
| Overhead/Clinic collaboration fee | $290.56 | $290.56 | $4.97 | $5.05 | $0.08 | $0.07 |
| ***Sub-total*** | ***$1757.32*** | ***$2935.52*** | ***$29.12*** | ***$48.98*** | ***$0.46*** | ***$0.68*** |
| **Summary** | ***$2542.30*** | ***$3725.00*** | ***$41.52*** | ***$61.60*** | ***$0.66*** | ***$0.85*** |
