# Supplementary material for: An Interactive Text Messaging Intervention to Improve Adherence to Option B+ Prevention of Mother-to-Child HIV Transmission in Kenya: Cost Analysis
Source: JMIR Mhealth Uhealth. 2020 Oct 2;8(10):e18351. doi: 10.2196/18351 (PMC7568211; doi:10.2196/18351)
Supplement: Multimedia Appendix 4 [file mhealth_v8i10e18351_app4.docx]

**Multimedia Appendix 4.** Sensitivity and scenario analysis: total annual costs and unit costs for beneficiaries.

| **Facility** | **Total costs and unit costs** | | |
| --- | --- | --- | --- |
|  | **Total annual cost (USD)** | **Cost per beneficiary**  **(USD)** | **Cost per contact**  **(USD)** |
| *Scenario 1* |  |  |  |
| Fixed | $3,542 | $6 | $0.10 |
| Variable | $19,801 | $36 | $0.53 |
| **Total** | **$23,344** | **$43** | **$0.63** |
| *Scenario 2* |  |  |  |
| Fixed | $3,542 | $6 | $0.10 |
| Variable | $13,542 | $25 | $0.36 |
| **Total** | **$17,084** | **$31** | **$0.46** |
