# Supplementary material for: An Interactive Text Messaging Intervention to Improve Adherence to Option B+ Prevention of Mother-to-Child HIV Transmission in Kenya: Cost Analysis
Source: JMIR Mhealth Uhealth. 2020 Oct 2;8(10):e18351. doi: 10.2196/18351 (PMC7568211; doi:10.2196/18351)
Supplement: Multimedia Appendix 5 [file mhealth_v8i10e18351_app5.docx]

**Multimedia Appendix 5.** Incremental cost-effectiveness ratios of two-way SMS versus no intervention in viral load suppression and adherence.

|  | **Control** | **Two-way** | **Difference** |
| --- | --- | --- | --- |
| Cost | $0 | $7084 | $7084 |
| VL suppression (N) | 55 | 66 | 11 |
| **ICER^*^** |  |  | **$644** |
| Cost | $0 | $7084 | $7084 |
| Adherence (N) | 58 | 73 | 15 |
| **ICER^*^** |  |  | **$472** |

Abbreviations: VL, viral load; ICER, incremental cost-effectiveness ratio.

**^*^** ICER is calculated by dividing the difference in total costs (incremental cost) by the difference in the chosen measure of health outcome or effect (incremental effect).
