# Supplementary material for: An Interactive Text Messaging Intervention to Improve Adherence to Option B+ Prevention of Mother-to-Child HIV Transmission in Kenya: Cost Analysis
Source: JMIR Mhealth Uhealth. 2020 Oct 2;8(10):e18351. doi: 10.2196/18351 (PMC7568211; doi:10.2196/18351)
Supplement: Multimedia Appendix 6 [file mhealth_v8i10e18351_app6.docx]

| **Parameter** | **Value** | **Source** |
| --- | --- | --- |
| Total annual incremental cost in facility A | $4111.21 | Project output |
| Total annual incremental cost in facility B | $2972.40 | Project output |
| Total annual incremental cost in two facilities | $7083.61 | Project cost estimate |
| Total beneficiaries in two-way group in two facilities | 115 | Project output |
| Total beneficiaries in control group in two facilities | 115 | Project output |
| % viral load suppressed in two-way group | 57% | Lester, 2010 |
| % viral load suppressed in control group | 48% | Lester, 2010 |
| Odds ratio of patients' medication adherence | 1.71 | Thakkar, 2016 |
| % adhere to ART in two-way group | 63% | Calculation |
| % adhere to ART in control group | 50% | Assumption |

**Multimedia Appendix 6.** Input parameters for incremental cost-effectiveness ratios of two-way SMS versus no intervention in viral load suppression and adherence.
